# Mitochondrial Genome and Nuclear Markers Provide New Insight into the Evolutionary History of Macaques

**DOI:** 10.1371/journal.pone.0154665

**Published:** 2016-05-02

**Authors:** Juan Jiang, Jianqiu Yu, Jing Li, Peng Li, Zhenxin Fan, Lili Niu, Jiabo Deng, Bisong Yue, Jing Li

**Affiliations:** 1 Key Laboratory of Bioresources and Ecoenvironment (Ministry of Education), College of Life Sciences, Sichuan University, Chengdu 610064 Sichuan, China; 2 Chengdu Zoo, Institute of Chengdu Wildlife, Chengdu 610081, China; 3 Sichuan Key Laboratory of Conservation Biology on Endangered Wildlife, College of Life Sciences, Sichuan University, Chengdu 610064, China; University of Florence, ITALY

## Abstract

The evolutionary history of macaques, genus *Macaca*, has been under debate due to the short times of divergence. In this study, maternal, paternal, and biparental genetic systems were applied to infer phylogenetic relationships among macaques and to trace ancient hybridization events in their evolutionary history. Using a PCR display method, 17 newly phylogenetically informative *Alu* insertions were identified from *M*. *assamensis*. We combined presence/absence analysis of 84 *Alu* elements with mitochondrial genomes as well as nuclear sequences (five autosomal genes, two Y chromosomal genes, and one X chromosomal fragment) to reconstruct a robust macaque phylogeny. Topologies generated from different inherited markers were similar supporting six well defined species groups and a close relationship of *M*. *assamensis* and *M*. *thibetana*, but differed in the placing of *M*. *arctoides*. Both *Alu* elements and nuclear genes supported that *M*. *arctoides* was close to the *sinica* group, whereas the mitochondrial data clustered it into the *fascicularis/mulatta* lineage. Our results reveal that a sex-biased hybridization most likely occurred in the evolutionary history of *M*. *arctoides*, and suggest an introgressive pattern of male-mediated gene flow from the ancestors of *M*. *arctoides* to the *M*. *mulatta* population followed by nuclear swamping. According to the estimation of divergence dates, the hybridization occurred around 0.88~1.77 mya (nuclear data) or 1.38~2.56 mya (mitochondrial data). In general, our study indicates that a combination of various molecular markers could help explain complicated evolutionary relationships. Our results have provided new insights into the evolutionary history of macaques and emphasize that hybridization might play an important role in macaque evolution.

## Introduction

Macaques represent one of the most successful primate radiations with 20–23 extant species in genus *Macaca*. According to Raaum et al. [1], macaques diverged from other members of the tribe *Papionini* approximately 9–10 million years ago (mya). Fossil data indicated that the genus arose about 7 mya in northern Africa and then the Asian macaque lineage began to appear around 5.5 mya [2,3]. With the exception of *M*. *sylvanus* in North Africa and Southern Europe, macaques are widely distributed in southern and eastern Asia [4], ranking second only to the world-wide distribution of humans among the extant primates. With such a variety of habitats and species that differ in ecology and external morphology, macaques are best known as a prime group for studies of species radiation and evolution as well as an important animal model in medical research. Thus it is important to elucidate the phylogeny of the extant taxa of macaques, which will contribute to our understanding of the evolutionary history of the genus as well as other primate radiations.

The relationships among macaques have been the focus of research [2,4–8] (S1 Table). Traditionally, based on male genitalia, Fooden [4] classified the genus into four species groups: *silenus-sylvanus* group, *fascicularis* group, *arctoides* group, and *sinica* group. Later, Delson [2] made a small modification by placing *M*. *arctoides* into the *sinica* group and *M*. *sylvanus* in a group by itself. Meanwhile, Groves [6] divided the genus into six species groups, arguing that Sulawesi macaques separated from the *silenus* group and formed their own group, and that *M*. *mulatta*, *M*. *fuscata* and *M*. *cyclopis* removed from the *fascicularis* group to form a new *M*. *mulatta* group. Recently, Zinner et al. [7] and Roos et al. [8] proposed seven species groups including three monotypic species groups (*M*. *sylvanus* group, *M*. *arctoides* group and *M*. *fascicularis* group) and four polytypic groups (Sulawesi *group*, *mulatta* group, *sinica* group and *silenus* group). Recent molecular studies were largely based on mitochondrial DNA (mtDNA) genes [9–14], autosomal genes [15,16], Y chromosomal sequences [16,17], and presence/absence polymorphism of *Alu* elements [18]. Despite some general consensus, substantial discordances detected on several branches, such as the relationships among closely related species within the same species groups (*sinica* group and *fascicularis* group) and the phylogenetic position of *M*. *arctoides*, remain unresolved. Furthermore, the short time of divergence and rapid radiation in the genus lead to introgression (i.e., introgressive hybridization) and gene flow among different species/species groups [16–20].

Different genetic markers, such as mobile elements and sequence data from the mitochondrial genome and nuclear genome, have their own unique characteristics in phylogenetic analysis. The complete mitochondrial genome has distinct advantages: a small sequence (~16 kb), a single and no-recombination locus and the maternally inherited, making it a better marker to reconstruct phylogenetic relationships than a single gene or partial genome sequences [21–24]. In addition, the mutation rate of mtDNA in primates was estimated five to ten times higher than that of the nuclear genome [25], indicating it is suitable to resolve relationships even among closely related species. On the other hand, the nuclear loci are essential as well, because they represent neutral and paternal inheritance. Particularly, non-coding intron sequences offer potentially powerful genetic markers, since they have a number of traits that are suitable for molecular phylogenies [26–29]. Another kind of nuclear marker, *Alu* elements, are thought to be promising tools to uncover phylogenetic relationships. They predominantly possess two remarkable properties: essentially homoplasy-free with identical by descent and unidirectional with the absence of the insertion being the ancestral state [30]. Besides, they are easy to genotype using only a PCR-based approach. Accordingly, *Alu* elements have been applied to address issues with respect to human population genetics [31–34] as well as numerous controversial primate phylogenetic relationships [35–37]. In addition, SNPs (single nucleotide polymorphisms) and microsatellites are also important nuclear markers that have been widely used for population structure and genetic diversity studies [38–42] due to the unique features of high polymorphism among populations or individuals. Both of them are seldom applied to phylogenetic analyses in previous studies. Given that different genetic markers provide different information, it is suggested that a more robust phylogeny could be derived from the combination of analyses on different genetic systems. Combined analyses of the different molecular markers have been successfully employed to uncover complicated evolutionary relationships and to ascertain reasons that are responsible for incongruent phylogenetic relationships [22,43–45].

Previous studies on macaque phylogenies were merely based on a single genetic system [9–15,18], or a combination of short mitochondrial genes and intron sequences [16,17]. Herein, a PCR display method was applied to *M*. *assamensis* to identify newly phylogenetically informative *Alu* loci for phylogenetic analyses. Then we examined presence/absence pattern of 84 mobile elements and compared the inferred phylogeny with those obtained from mitochondrial data (10,832 bp each species) and nuclear sequence data of autosomes, Y and X chromosomes (9,465 bp each species). By combining these different markers systems, we attempted to resolve the controversial evolutionary relationships in macaque phylogeny, particularly the position of *M*. *arctoides*, and to trace ancient hybridization events in the macaque evolutionary history.

## Materials and Methods

### Samples collection and DNA extraction

Species analyzed in this study are listed in S2 Table including eight macaque species and *Papio hamadryas* as an outgroup. According to the records in captivity, all macaques have no opportunity to interbreed, and all individuals used in the study did not involve artificial interbreeding or hybridization. The Chengdu Institute of Biology Animal Use Ethics Committee approved this study on the phylogeny of macaques on the basis of the mitochondrial genome and nuclear data. The blood samples were obtained from captive macaques, which solely lived in large steel cages (height: 10m, length: 7.5, and width: 5m). Before drawing blood, these monkeys were anethetised with an intramuscular injection of mixed ketamine (10 mg/kg) and xylazine 0.25–2.0 mg/kg). During the process, animal’s body temperatures, respiration and heart rate were monitored to alleviate suffering. One mL of blood was drawn and stored in disposable vacuum blood vessels with EDTA-K2. These monkeys were not injured throughout the procedure and immediately released after recovery from sedation. All the operations were carried out by professional veterinarians. Total genomic DNA was extracted from whole blood using standard phenol/chloroform methods [46].

### *Alu* insertion loci identification and genotyping

Phylogenetically informative *Alu* loci from *M*. *assamensis* were identified by the PCR assay following the described methods [18,47]. Genomic DNA of *M*. *assamensis* was digested by a restriction enzyme *Nde*I (TaKaRa, China), and then ligated with double stranded linkers (S3 Table). For the first round, ligation products were amplified by using the LNP primer and a rhesus *Alu* specific primer YbI or YdI in order to obtain partial *Alu* sequences with the flanking unique sequences. The second round of PCR was performed using *Alu* YbII or YdII in order to obtain more specific amplicons. The amplicons were cloned into the pMD19-T vector (TaKaRa, China) and then transformed into DH5α competent cells. Positive clones were randomly screened and identified using the primer M13-47, sequencing on ABI 3730 Genetic Analyzer (Applied Biosystems). Sequences with a target *Alu* element and at least 100 bp of flanking sequences were used as queries in the BLAT [48] searching against rhesus macaque genome to discover the homologous position of insertion. Polymorphic *Alu* insertions that were present in the Assamese macaque but absent in the rhesus macaque genome were potentially phylogenetically informative and used for further phylogenetic reconstructions.

After the computational search, primers for the candidate loci were designed in flanking sequences using Primer3 [49] based on the reference genome. All primers were firstly tested for PCR amplifications with a temperature gradient (48–60°C) on a template consisting of Assamese and rhesus macaques to verify the most appropriate annealing temperature. Then these newly identified *Alu* loci (S4 Table), together with 67 previously published loci (S5 Table), which were obtained from *M*. *mulatta* and *M*. *fuscata* [50], and from *M*. *arctoides*, *M*. *thibetana*, *M*. *nemestrina* and *M*. *fascicularis* [18,51], were further genotyped for the presence/absence pattern in all specimens. The detailed information on each locus, including primer sequences, chromosomal locations, annealing temperatures, PCR product sizes, and amplification results of studied species was described in S4 and S5 Tables. Presence of an insertion was designated as “1”, absence as “0”, and missing data as “?”. The newly identified *Alu* loci from *M*. *assamensis* were deposited in GenBank with the following accession numbers KU612224-KU612231; KU645892-KU645899; and KU641401.

### PCR amplification and sequencing

Complete mitochondrial genomes of the nine investigated species were obtained from GenBank (S6 Table), and the 12 mitochondrial protein coding genes (except for *ND6* gene) were used for analyses. We also analyzed nuclear sequence data including five autosomal genes (*ALB3*, *IRBP3*, *TNP2*, *TTR1*, and *vWF11*) [22], two Y chromosomal genes (*SRY* and *TSPY*) [17], and one X chromosomal fragment (*Xq13*.*3)* [52]. We amplified these nuclear genes from each of the macaque species according to the primers in S3 Table and downloaded available nuclear sequences from GenBank (S6 Table). PCR amplification for each gene was set up in a 25μL reaction volume containing at least 25 ng total DNA, 200 nM of each primer, 200 μM dNTPs, 2.5 μL of 10**×**PCR buffer, and 2.5 U Taq DNA polymerase (TaKaRa, China). PCR amplification was carried out at 94°C for 5 min, 35 cycles of denaturation at 94°C for 1 min, annealing at varying temperatures for 1 min, extension at 72°C for 1 min, and a final extension step at 72°C for 10 min. PCR products were checked on 2% agarose gels, visualized using UV fluorescence (Bio-Rad, Hercules, CA), and sequenced on ABI 3730 Genetic Analyzer (Applied Biosystems). The newly obtained sequences were deposited in GenBank with accession numbers KT356221-KT356259.

### Phylogenetic analyses

For the *Alu* elements, phylogenetic reconstruction was implemented by an exhaustive search with 10,000 bootstrap replications via the PAUP* 4.0b10 software [53] using Dollo parsimony analysis. The sequence data were initially aligned using MEGA 5.2.2 [54] with default settings. Subsequently, poorly aligned positions and indels were removed by eye. Multiple analyses were then performed on the combined 12 mitochondrial protein coding genes and concatenated nuclear dataset. To test whether different nuclear data can be combined, partition homogeneity tests were performed in PAUP with 1000 replicates. Simultaneously, in order to compare the results from different genetic markers, we also performed analyses on five autosomal loci, two Y chromosomal genes, and one X chromosomal region, respectively. Phylogenetic analyses of all datasets were implemented using PAUP* 4.0b10 [53] for MP analyses, using MrBayes v3.1.2 [55] for Bayesian inference (BI) as well as using PHYML[56,57] for ML analyses with 500 bootstrap replications. For MP analyses, the reliability of the clades in phylogenetic trees was assessed by bootstrap probabilities (BSP) computed using 1000 replicates with 20 random additional sequencing replicates for each bootstrap replicate. Alternatively, for ML and Bayesian algorithms, the optimal nucleotide substitution model were chosen using the Akaike Information Criterion (AIC) as implemented in jModeltest 2.1 [58]. For Bayesian reconstructions, relative support of internal nodes was assessed by Bayesian posterior probabilities (BPP) analyses. Two separate runs were performed with four Monte Carlo Markov Chains with the default temperature of 0.1. Four repetitions were run for 1000,000 generations with tree and parameter sampling occurring every 100 generations. The first 25% of samples were discarded as burn-in, leaving 75,001 trees per run. Posterior probabilities for each split were calculated from the posterior density of trees.

### Divergence age estimation

A Bayesian MCMC method was applied to estimate divergence times within the genus *Macaca* by using software BEAST v1.7.5 [59]. As calibration, we used the fossil-based divergence between African and Asian lineages, which split about 5.5 mya [2,3,16] as well as the divergence between *Papio* and *Theropithecus gelada*, which split ~ 4 mya [60]. In BEAST, a correlated lognormal model of lineages variation and a Yule process for branching rates were implemented in each analysis. Divergence times were estimated for the nuclear dataset and the combined 12 mitochondrial genes with a mean of 5.5 mya and a standard deviation (SD) of 0.4 mya for the separation of *M*. *sylvanus* and Asian macaques as well as with a mean of 4.0 mya and SD of 0.5 mya for the *Papio* and *T*. *gelada*. For the MCMC analysis, two replicates were run for 1000,000 generations with tree and sampling occurring every 1000 generations. The log output files were carried out by the program Tracer [61]. 10% of the trees were discarded as burn-in, and the remaining was assessed by TreeAnnotator v1.7.5 within the BEAST package to obtain a consensus tree, which was visualized with Figtree v1.4.0 [62].

## Results

### Phylogenetic analyses of *Alu* elements

Combining a computational approach and a PCR display method, we identified phylogenetically informative *Alu* loci from *M*. *assamensis*. A total of 154 clones were randomly selected for sequencing analyses. The results indicated that 70 sequences contained no repetitive elements or elements beyond the *Alu*Y subfamily. The remaining 84 sequences with target *Alu*Y elements were further applied to the BLAT search. Among those 84 sequences, we failed to identify the homologous locations of 17 sequences owing to short flanking sequences. After the BLAT search, an additional 40 *Alu* loci were found to be shared by the rhesus macaque genome and the Assamese macaque genome, indicating they were probably not phylogenetically informative. We identified 27 phylogenetically informative *Alu* loci in *M*. *assamensis*. An additional 10 insertions were rejected because of failures of primer designing or genotyping. Ultimately, 17 novel insertions identified from Assamese macaque were amplified in all samples and were used for further phylogenetic reconstructions.

**Fig 1:**
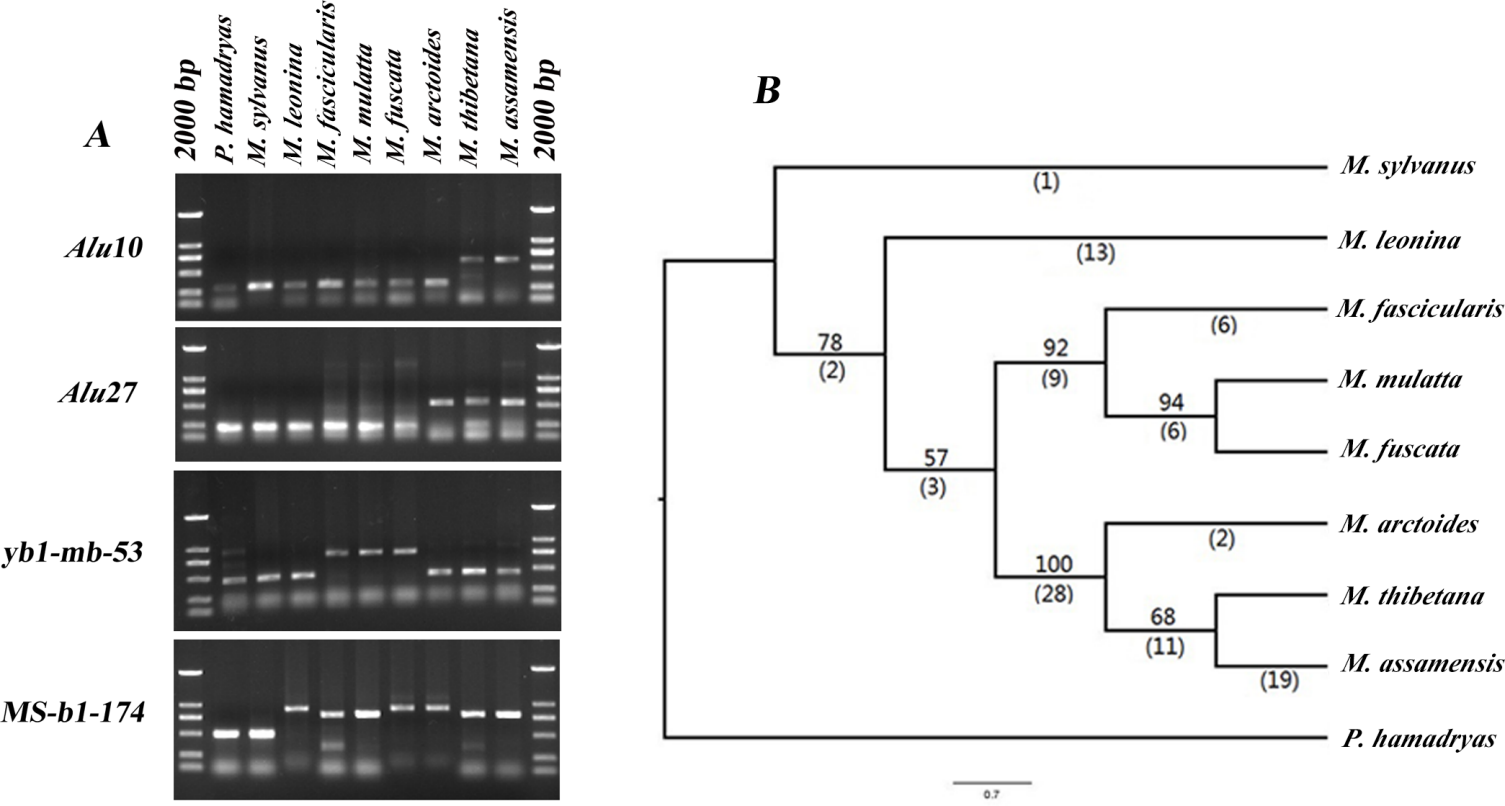
Phylogenetic relationships among macaques based on *Alu* elements. (A) PCR amplification analysis of *Alu* insertion polymorphisms in *Macaca*. The locus *Alu* 10 is an *Alu* insertion specific to the *sinica* group. The locus *Alu* 27 is an *Alu* insertion shared by the *sinica* and the *arctoides* groups. The locus yb1-mb-53 is an *Alu* insertion shared by the *fascicularis* and the *mulatta* groups. The locus MS-b1-174 is an *Alu* insertion clustering the *sinica/arctoides* and the *fascicularis/mulatta* lineages. (B) Macaque phylogenetic tree derived from 84 *Alu* insertion loci polymorphisms. The amplification patterns of the *Alu* insertions were used to construct a Dollo parsimony tree of macaque phylogenetic relationships using *P*. *hamadryas* as outgroup in PAUP*4.0b10. The numbers above the branches indicate the percentage of bootstrap replicates (1000 iterations) producing trees including that node. The numbers below the branches indicate the number of unambiguous insertions supporting each node.

Combined with 67 previously reported *Alu* loci, a total of 84 loci were genotyped the presence/absence in eight macaque species and one outgroup. Four examples of gel electrophoresis patterns of amplification results are shown in Fig 1A, and the detailed information on gel electrophoresis of all *Alu* insertions in our study was shown in S1 Fig. Sixty-three of the 84 *Alu* loci were found to be parsimony informative generating a single most parsimonious tree [Fig 1B, consistency index (CI) = 0.785; homoplasy index (HI) = 0.215; retention index (RI) = 0.770]. The topology of the tree clearly defined four main clades and six species groups: *sylvanus* group (*M*. *sylvanus*), *silenus* group (*M*. *leonina*), *arctoides* group (*M*. *arctoides*), *sinica* group (*M*. *assamensis* and *M*. *thibetana*), *fascicularis* group (*M*. *fascicularis*), and *mulatta* group (*M*. *mulatta* and *M*. *fuscata*), which were consistent with the most recent classification [7, 8]. *M*. *sylvanus* was in a basal position as a sister to all Asian macaques. Within the Asian macaque lineage, thirteen *Alu* insertion loci supported that *M*. *leonina* diverged first forming a sister clade to other four species groups (BSP = 72%). Whereas 28 unambiguous insertions clustered *M*. *assamensis*, *M*. *thibetana* and *M*. *arctoides* together with high bootstrap values (BSP = 100%). Another 11 insertions were shared by *M*. *assamensis* and *M*. *thibetana* indicating a close relationship between them, while *M*. *arctoides* formed a sister clade to them (BSP = 68%). Within the *fascicularis/mulatta* lineage, six *Alu* insertion loci supported *M*. *fascicularis* diverging first, and *M*. *mulatta* and *M*. *fuscata* shared a close relationship which was supported by six *Alu* insertion loci.

### Phylogenetic analyses of nuclear dataset

**Fig 2:**
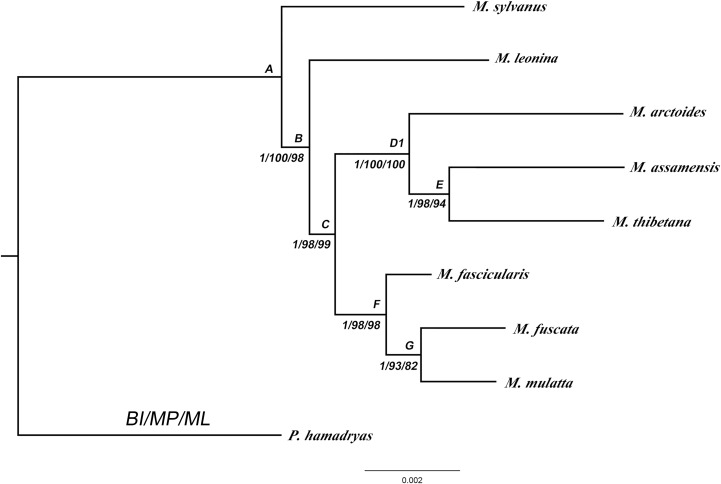
Molecular phylogenetic tree derived from nuclear data using Bayesian, MP and ML analysis. The numbers are Bayesian posterior probabilities (BPP) and bootstrap support (BSP). The A-G besides the nodes refers to divergence times shown as in Table 2.

Next, phylogenetic reconstructions were performed on the combined nuclear sequences of five autosomal loci, two Y chromosomal genes and one X chromosomal region. Partition homogeneity tests revealed no significant difference in their evolutionary history (Y chromosomal loci combined: P = 0.2471; autosomal loci combined: P = 0.1305; all nuclear loci combined: P = 0.3021). The concatenated nuclear datasets were consisted of 9,465 nucleotide positions. The best fit model of nucleotide substitution for the concatenated dataset was TVM+I (Table 1). The CI (0.9636) and RI (0.8171) of the nuclear dataset were higher than those of *Alu* elements and mitochondrial data, which indicated less homoplasy of the nuclear genes. Phylogenetic trees obtained from BI, MP and ML analyses yielded identical and significantly supported branching patterns (BPP = 1, BSP> 93%, BSP>94%) (Fig 2). Only the *M*. *fuscata/mulatta* clade gained relatively weak bootstrap values (ML: 82%). In principal, the resultant tree topology was identical to the *Alu* element-based phylogeny.

**Table 1:** Characterization of nuclear loci and mitochondrial genome for phylogenetic analyses.

Locus	Length	Inform-ative	Variable	CI	RI	Best model	Alpha
ALB3	809	2	29	0.9688	0.8750	HKY	N/A
IRBP3	1561	19	76	0.9796	0.9545	TVM	N/A
TNP2	815	10	79	0.9663	0.8000	GTR+G	0.1464
TTR1	743	1	29	0.9818	0.7857	TVM+I	N/A
vWF11	945	3	42	1.0000	1.0000	TrN	N/A
**Com autosome**	**4973**	**35**	**255**	**0.9597**	**0.7105**	**TVM+I+G**	**0.1459**
**X chromosome**	**1498**	**6**	**21**	**1.0000**	**1.0000**	**TVM+I+G**	**N/A**
SRY	770	6	23	1.0000	1.0000	TVM	N/A
TSPY	2224	23	75	0.9870	0.9688	GTR	N/A
**Com Y chromosome**	**2994**	**29**	**98**	**0.9892**	**0.9722**	**GTR+I+G**	**N/A**
**Com nuclear genome**	**9465**	**70**	**374**	**0.9636**	**0.8171**	**TVM+I+G**	**0.0774**
ATP6	678	132	258	0.6797	0.4739	TrN+G	0.3200
ATP8	198	42	81	0.7016	0.5375	TrN+I	N/A
COX1	1539	241	436	0.6539	0.4404	HKY+G	0.1900
COX2	681	108	189	0.6472	0.4577	TIM+G	0.1330
COX3	783	116	243	0.7378	0.5309	TPM2uf+G	0.3310
CYTB	1141	184	344	0.6619	0.4388	TrN+G	0.1710
ND1	948	142	277	0.6857	0.4923	HKY+G	0.2270
ND2	1038	155	325	0.7231	0.5092	TrN+G	0.2600
ND3	345	59	121	0.7500	0.5591	TrN+I	N/A
ND4	1378	252	477	0.6576	0.4325	TrN+I	N/A
ND4L	294	47	100	0.7034	0.4756	TrN+I	N/A
ND5	1809	281	585	0.7064	0.4667	TPM2uf+G	0.3100
**Com Mt**	**10832**	**1759**	**3436**	**0.6822**	**0.4610**	**TIM2+G**	**0.2470**
**Total datasets**	**20297**	**1829**	**3810**	**-**	**-**	**-**	

CI: consistency index; RI: retention index; Best model: best fitting model under the Akaike information criterion; Alpha: gamma distribution shape parameter; Mt: mitochondrial genome.

We also performed phylogenetic analyses based on autosomal loci, Y chromosomal genes, and X chromosomal fragment. The best fit models of nucleotide substitution for the three datasets were TVM+G, GTR+I and TVM (Table 1), respectively. The trees estimated based on different molecular markers are shown in S2 Fig. The results appeared similar to the *Alu* element-based tree and the combined nuclear tree, but differed in several nodes. First, *M*. *fuscata* was closely related to *M*. *mulatta* in the *Alu* elements tree, Y and X chromosomal trees, and the combined nuclear genes tree, while, in the autosomal tree, it formed a sister clade to other four species groups with margin of support values (BPP = 0.86, BSP = 65%, BSP = 66%). Second, the position of *M*. *arctoides* was unstable in different nuclear trees. Both autosomal and Y chromosomal trees supported that *M*. *arctoides* diverged earlier than the split of *M*. *thibetana* and *M*. *assamensis*, which was consistent with the combined nuclear tree and the *Alu* element-based tree, while X chromosomal tree showed *M*. *arctoides* was closer to *M*. *assamensis* than to *M*. *thibetana*. Unexpectedly, the Y chromosomal tree nested *M*. *sylvanus* within the Asian macaques, and then clustered with the *sinica/arctoides* lineage (BPP = 0.21, BSP = 51%, BSP = 70%) contradicting to other nuclear genes, *Alu* elements, and mitochondrial topologies.

### Phylogenetic analyses of mitochondrial dataset

**Fig 3:**
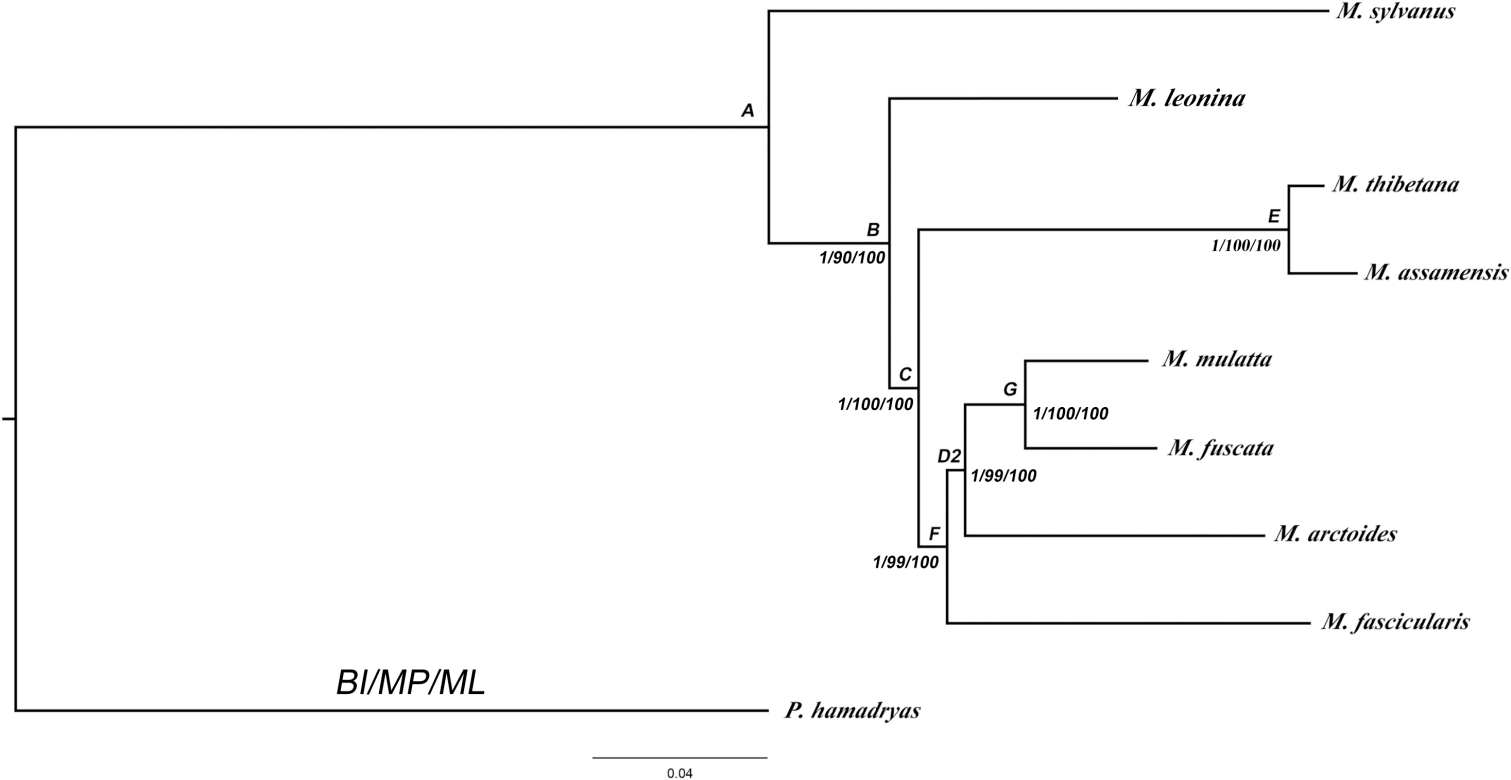
Molecular phylogenetic tree obtained from mitochondrial genome using Bayesian, MP and ML analysis. The numbers are Bayesian posterior probabilities (BPP) and bootstrap support (BSP). The A-G besides the nodes refers to divergence age shown as in Table 2.

We did not combine mitochondrial and nuclear data due to differences in effective population size, dispersal rate, and mode of inheritance [63,64]. The combination of 12 mitochondrial protein coding genes included 10,832 aligned nucleotide positions. The best fit model of nucleotide substitution was TIM2+G (Table 1). The topology of mitochondrial data tree (Fig 3) was congruent with those obtained from mobile elements and nuclear sequence data with the exception of the position of *M*. *arctoides*. The mitochondrial data tree unambiguously clustered *M*. *arctoides* into the *fascicularis/mulatta* lineage. Particularly, it showed a closer relationship to the *mulatta* group than to the *fascicularis* group (BPP = 1, BSP = 99%, BSP = 100%), whereas both the *Alu* elements and the nuclear genes supported a close relationship of *M*. *arctoides* to the *sinica* group (*M*. *thibetana* and *M*. *assamensis*).

### Divergence age estimation

Divergence age estimation based on nuclear genes and the mitochondrial genome was summarized in Table 2 and S3 Fig. The nuclear data estimated that the African and Asian lineages separated ~5.36 mya. In the Asian lineage, an initial split occurred at ~4.32 mya, which led to a clade containing *M*. *leonina* and a clade consisting of the *fascicularis/mulatta* lineage and the *sinica/arctoides* lineage. These two lineages successively diverged from each other around 3.08 mya. Within the *fascicularis/mulatta* lineage, *M*. *fascicularis* diverged first around 1.79 mya, and then *M*. *fuscata* separated from *M*. *mulatta* at ~0.88 mya. Speciation and divergence of the *sinica/arctoides* lineage occurred almost at the same time with the *fascicularis/mulatta* lineage. Within the *sinica* group, *M*. *arctoides* represented an early divergence at ~1.77 mya in this lineage, while *M*. *thibetana* and *M*. *assamensis* represented the most recent separation at ~0.58 mya. However, the divergence estimations based on the mitochondrial genome generally were earlier than the estimations based on nuclear data. The mitochondrial tree inferred that the divergence date between *fascicularis/mulatta* lineage *and sinica* lineage was ~3.81 mya. Within the *fascicularis/mulatta* lineage, *M*. *fascicularis* diverged first at ~2.92 mya. The mitochondrial tree placed *M*. *arctoides* in the *fascicularis/mulatta* lineage and indicated that the divergence of *M*. *arctoides* occurred ~2.56 mya, which was earlier than the split of *M*. *mulatta* and *M*. *fuscata* (~1.38 mya). Both the nuclear and mitochondrial data supported that the most recent divergence occurred between *M*. *thibetana* and *M*. *assamensis* at no more than 1.0 mya.

**Table 2:** Divergence date estimations based on the nuclear genes and mitochondrial genome.

Node	Divergence dates (MYA)	
	nuclear genes	mitochondrial genome
*Macaca*—*Papio/Theropithecus*	6.58 (4.91–10.6)	9.35 (4.75–11.4)
*Papio*—*Theropithecus*	3.83 (2.45–4.76)	3.89 (3.14–4.66)
(A) African—Asian macaque	5.36 (4.84–6.01)	5.38 (4.69–6.04)
(B) *silenus* group	4.32 (1.98–5.79)	4.83 (4.24–5.55)
(C) *sinica—fascicularis/mulatta* lineage	3.08 (0.99–5.11)	3.81 (3.23–4.55)
(D1) *M*. *arctoides—M*. *thibetana/M*. *assamensis*	1.77 (0.27–3.75)	-
(D2) *M*. *arctoides—mulatta* group	-	2.56 (1.75–3.51)
(E) *M*. *thibetana—M*. *assamensis*	0.58 (0.02–2.45)	0.54 (0.01–0.71)
(F) *fascicularis—mulatta* group	1.79 (0.40–3.82)	2.92 (0.18–3.60)
(G) *M*. *mulatta—M*. *fuscata*	0.88 (0.13–2.40)	1.38 (0.04–1.80)

## Discussion

### Combination of different genetic systems to infer macaque phylogeny

Different types of genetic markers are subjected to a unique stress from biological and behavioral conditions reflecting different evolutionary signals. The mitochondrial genome, Y chromosome, and autosome represent maternal, paternal, and bi-parental lineage, respectively. Single genetic marker can only reflect one kind of evolutionary pattern, making it difficult to discuss comprehensive analysis. However, multiple genetic systems can provide helpful information in phylogenetic analyses [16,22,43–45]. By combining presence/absence pattern of *Alu* elements with autosomal, Y chromosomal, X chromosomal sequences, and the mitochondrial genome, our study provides comprehensive insights into the evolutionary history of macaque species. The phylogenetic trees and the estimated divergence ages from different datasets are broadly in line with those reported in previous studies [16,18,23]. Compared with previous macaque phylogenetic studies, most relationships have been resolved and obtained stronger supported by *Alu* elements and sequence data in our study. Although we did not include the Sulawesi macaques, other six species groups (*sylvanus*, *silenus*, *sinica*, *arctoides*, *mulatta*, and *fascicularis*) has been clearly defined in the genus *Macaca*, which are generally in agreement with the results of Zinner et al. [7] and Roos et al. [8]. *M*. *sylvanus* is a sister taxon to all the Asian lineages, Within the Asian macaques, *M*. *leonina* in the *silenus* group separated first followed by the two sister clades, the *sinica/arctoides* and the *fascicularis/mulatta* lineages. They finally divided into the extant four species groups. Our study further supports two close relationships within this genus: *M*. *assamensis* and *M*. *thibetana* as well as *M*. *mulatta* and *M*. *fuscata*. The study also revealed several significant discrepancies among genetic systems and inferred sex-biased introgression or hybridization among ancestral macaque lineages such as the observed phylogenetic incongruences with respect to *M*. *arctoides* (see below for more details). In conclusion, our study suggested that the combination of maternally, paternally, and bi-parentally inherited markers along with the combination of sequence data with presence/absence patterns of mobile elements proved to be an adequate and reliable phylogenetic approach, particularly for revealing hybridization events.

### The phylogenetic position and evolutionary history of *M*. *arctoides*

Based on morphological characters, such as reproductive organs [65,66], hair growth patterns [5,66,67], dentition and cranium structure [2,66], and allozyme frequencies [65,66,68], previous studies suggested that *M*. *arctoides* was closely associated with the *sinica* group. However, the molecular data with respect to the position of *M*. *arctoides* is less concordant. Phylogenetic analyses based on autosomal loci [15–17], Y chromosomal genes [16,17], and *Alu* elements [18] consistently agreed with the morphological studies in assigning *M*. *arctoides* into the *sinica* group. However, mitochondrial genes indicated that *M*. *arctoides* should be ascribed to the *fascicularis* group [9–14]. Even within the same species group, the evolutionary relationship of *M*. *arctoides* to other macaques remained unstable. In the present study, 28 *Alu* insertions were shared by *M*. *arctoides*, *M*. *thibetana*, and *M*. *assamensis*, strongly confirming a close relationship among them. The close relationship of *M*. *arctoides* to the *sinica* group was further supported by the autosomal, Y chromosomal, and X chromosomal trees. With respect to the evolutionary relationship among the three macaques, both the nuclear genes and *Alu* elements consistently inferred that *M*. *arctoides* was a distinct species, which diverged earlier than the split of *M*. *thibetana* and *M*. *assamensis*. And the inferred relationship was strongly supported with high bootstrap values, whereas the X chromosomal tree lumps *M*. *arctoides* with *M*. *assamensis*, to the exclusion of *M*. *thibetana* with a marginal support value. According to similar morphological characteristics such as a relatively short tail and big body shape, many previous studies have suggested that *M*. *arctoides* was probably derived from a *M*. *thibetana-*like ancestor [18,66] or an ancestor closely related to proto-*M*. *thibetana/assamensis* [15–17]. However, our results disagreed with these hypotheses suggesting an early divergence of *M*. *arctoides*, and the molecular clock estimated that the divergence of *M*. *arctoides* occurred at ~1.77 mya. Further investigation should be employed with more sufficient samples from other *sinica* group species and including more genetic markers from the whole genomes to ascertain the evolutionary relationships among these closely related species.

Similar to previous mitochondrial studies, our mitochondrial genome tree contradicted the nuclear genes and the *Alu* elements-based tree on the position of *M*. *arctoides*. The complete mitochondrial genome demonstrated convincingly a close association of the stump-tailed macaque to the *mulatta* group instead of the *sinica* group. Based on one or several mtDNA genes, Morales and Melnick [11] and Li and Zhang [12,13] suggested *M*. *arctoides* was closer to *M*. *fascicularis* than to *M*. *mulatta*, whereas Tosi et al. [17] inferred it was closer to *M*. *mulatta* than to *M*. *fascicularis*. Our results associated *M*. *arctoides* with the *mulatta* group, which was consistent with Tosi et al. [17]. The estimated divergence age of *M*. *arctoides* was around 2.56 mya, after *M*. *fascicularis* had separated (~2.92 mya) and before the split of *M*. *fuscata* and *M*. *mulatta* (~1.38 mya). The divergence age estimations from the mitochondrial genome were slightly earlier than that from the nuclear dataset, most likely due to the relatively faster evolutionary rate of mitochondrial genes than nuclear genes [25].

Incongruent phylogenetic relationships among genes are common in phylogenetic analyses and could be explained by insufficient data, homoplasy, and incomplete lineage sorting (ILS) or hybridization [69–72]. In order to resolve which of these possibilities have been responsible for the discordances on the position of *M*. *arctoides*, we combined phylogenetic analyses based on maternal, paternal, and biparental genetic systems. First of all, inadequate data are not likely a problem, since both the base pairs of the nuclear dataset and mitochondrial genome are more than 9000 pairs. Similarly, homoplasy is also unlikely, because CI and RI of the *Alu* elements (0.785, 0.770), the nuclear dataset (0.964, 0.817) and the mitochondrial genome (0.682, 0.461) are relatively high. In addition, ILS cannot be the explanation for the incongruent phylogenetic relationships among genes because such limited divergence failed to account for the extensive similarities. Furthermore, mtDNA is less likely to show ILS, since it has a smaller effective population size which argues strongly against an ILS hypothesis. Although we cannot completely rule out that ILS may have had an effect by the combination of analyses on different genetic systems, a more likely reason for the detected discordances from nuclear and mitochondrial phylogenies is ancient hybridization between the closely related macaque species.

We further conducted comparative analysis of phylogenies generated from different genetic systems to reveal the potential hybridization pattern in *M*. *arctoides*. Tosi et al. [16,17] pointed out that *M*. *arctoides* was probably a hybrid taxon, originating from interbreeding proto-*sinica* species and proto-*fascicularis* species. Based on a large-scale application of *Alu* elements, the study of Li et al. [18] allied with the hybrid origin hypothesis. Based on the more sufficient datasets from maternal, paternal, and biparental genetic systems, we confirm the previous studies finding an ancient hybridization in *M*. *arctoides*. Nevertheless, *M*. *arctoides* is not of hybrid origin, and the hybridization occurred after it diverged from other *sinica* species. In particular, our results suggested that the hybridization in *M*. *arctoides* was not bidirectional but was more likely a male-mediated introgression followed by nuclear swamping.

**Fig 4:**
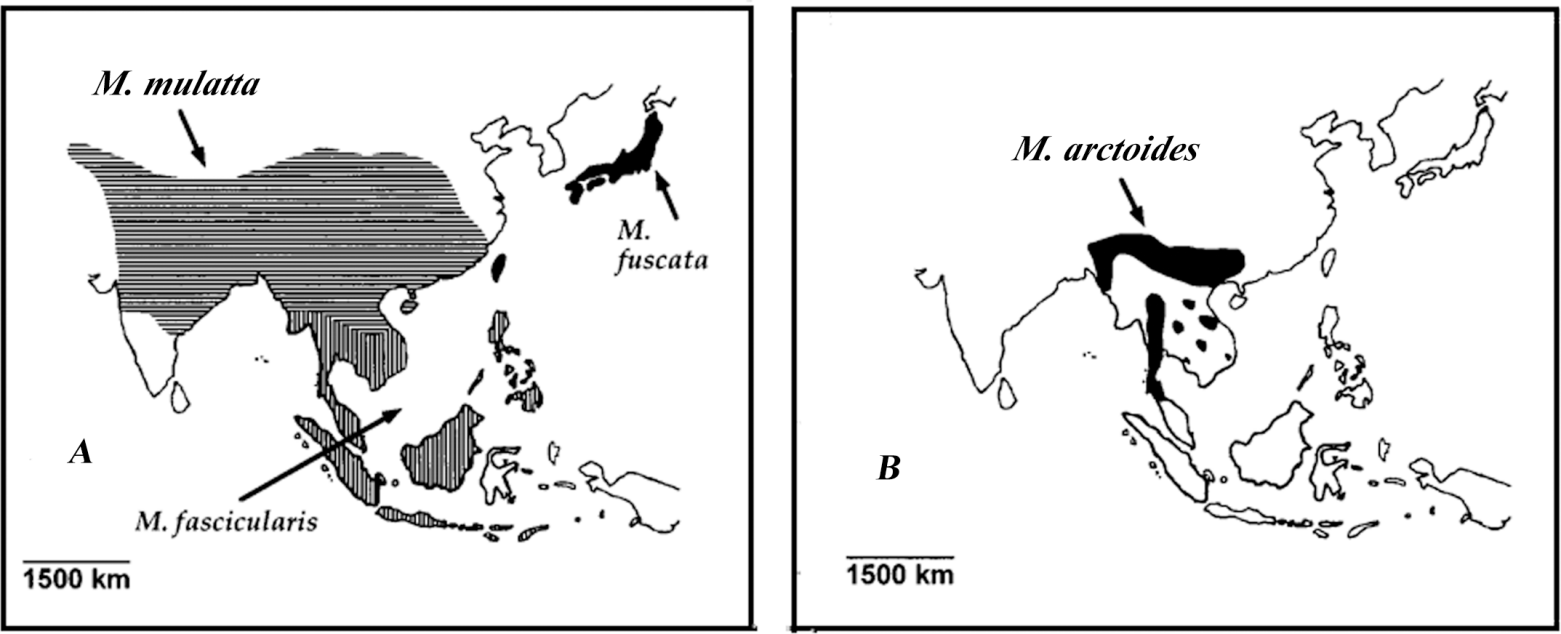
Distribution maps of *M*. *mulatta* and *M*. *arctoides*. A refers to *M*. *mulatta*, and B represents *M*. *arctoides*. Distribution contours of individual species are according to Corbet and Hill [74].

**Fig 5:**
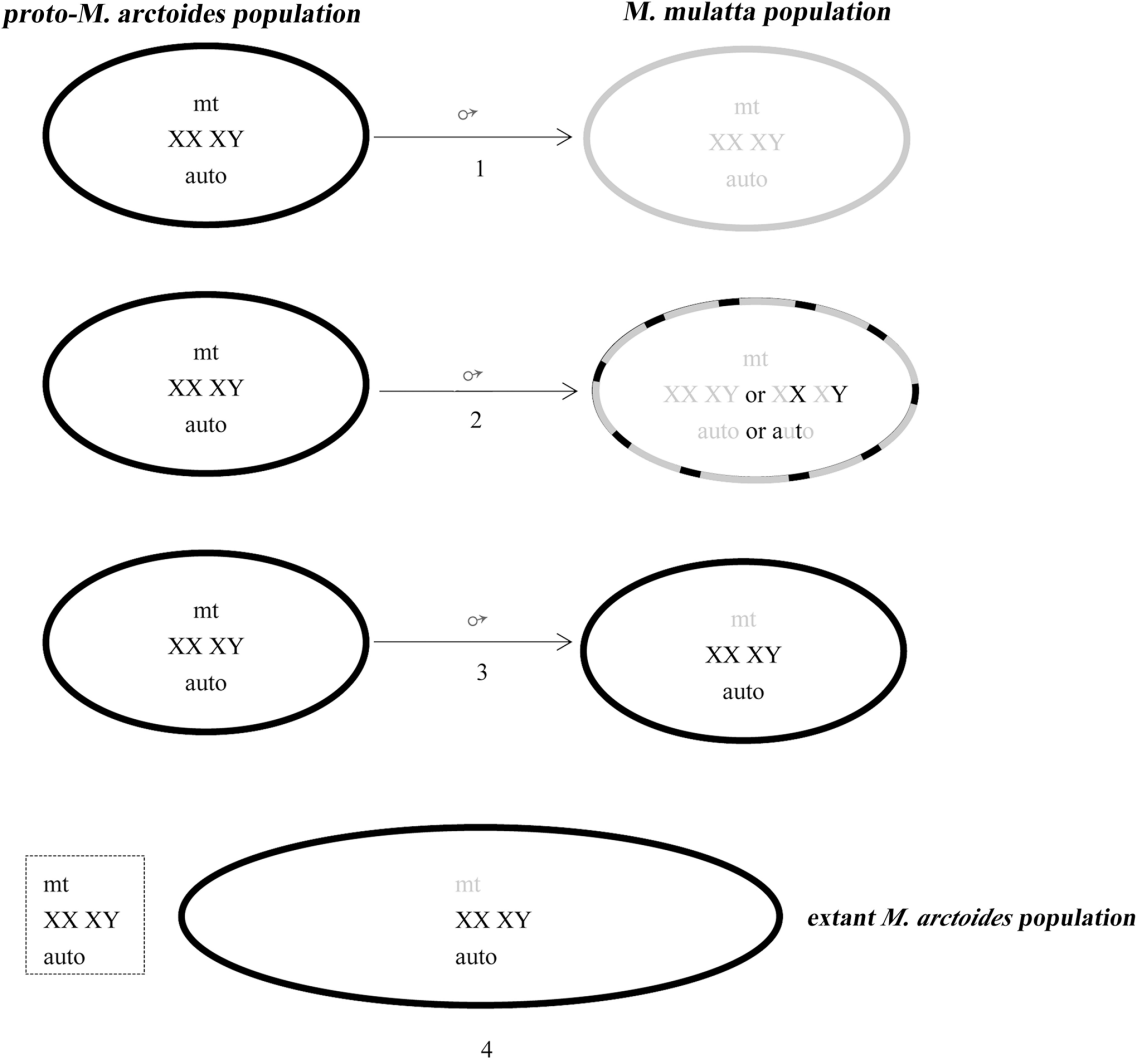
Male introgression and nuclear swamp. The proto-*M*. *arctoides* population is characterized by a black mitochondrial genome (mt), and black sex chromosomes (XY) and autosomes (auto). The *M*. *mulatta* population covers a grey mitochondrial (mt), grey sex chromosomes (XX), and autosome. (1) Given male migration and female philopatry, the males from population proto-*M*. *arctoides* invade population *M*. *mulatta* and produce hybrid offspring carrying grey mt but a half black sex chromosome and autosome. (2) The female hybrids from population *M*. *mulatta* backcrossed with males of population proto-*M*. *arctoides*. (3) If this continued over generations, the frequency of population *M*. *mulatta* genetic signals on the grey sex chromosome and autosome would significantly decrease. The grey nuclear genome will be swamped by black nuclear genome until it is completely replaced. In contrary, the grey mitochondrial genome will remain in population *M*. *mulatta*. (4) The extant *M*. *arctoides* population only has one grey mitochondrial haplotype and one black nuclear genome. The black mitochondrial genome (dotted line in the square) disappeared probably due to bottleneck during glaciations period. (The Fig imitates Zinner et al. [75]).

Given the high levels of male dispersal and extreme female philopatry in macaques, the most likely outcome of hybridization might be male-mediated introgression. According to the distribution and speciation of *M*. *arctoides* and *M*. *mulatta* (Fig 4), we assume that after the speciation of *M*. *arctoides*, the ancestors of *M*. *arctoides* pushed into South Asia because of the invasion of savanna and dry grassland [73] where the males of *M*. *arctoides* dispersed into the *M*. *mulatta* population, thus hybridization between them could have happened. The studies of Tosi et al. [16,17] also suggested that extensive hybridization events could have occurred between early *sinica* and *fascicularis* group members in a Pleistocene forest refugium. If the male-mediated gene flow continued over generations, and the hybrid offspring had backcrossed with male *M*. *arctoides*, the frequency of *M*. *mulatta* genetic signals on the sex chromosomes and autosomes would have been significantly reduced. The nuclear genome of *M*. *mulatta* thus have been ‘‘swamped” by that of *M*. *arctoides* in the hybrid population until it was completely replaced. In contrast, the original *M*. *mulatta* mitochondrial genome would stay in the population (Fig 5). The hybrid population gave rise to a unique genetic entity of the extant *M*. *arctoides* retaining a mitochondrial genome of the origin of rhesus macaque but autosomes, Y and X chromosomes of the *sinica* group. The divergence date estimation indicated that the introgression occurred 0.88~1.77 mya based on nuclear genes, or 1.38~2.56 mya based on mitochondrial genome, before the split of *M*. *mulatta*/*fuscata* and the split of *M*. *thibetana*/*assamensis*.

Although the male-mediated introgression hypothesis was in agreement with our extensive analyses on maternal, paternal, and bi-parental molecular markers, there exists another hybridization hypothesis. For instance, a mitochondrial capture from *M*. *mulatta* to *M*. *arctoides* also could result in a similar discrepancy between the mitochondrial genome and nuclear genome. This hypothesis assumes that females of *M*. *mulatta* dispersed into the population of *M*. *arctoides* and hybridized with males of *M*. *arctoides*, whereas no backcrossing with the invading *M*. *mulatta* took place. If the process continued for generations, the nuclear genome of the invaded *M*. *arctoides* population would barely change but absorb the mitochondrial genome of *M*. *mulatta*. However, mitochondrial capture has never been suggested in macaque species due to the female philopatry and male dispersal characteristics. Thus we suggest that the hybridization hypothesis in *M*. *arctoides* would benefit from more extensive genome-wide investigations.

### Other gene tree discordances in macaque phylogeny

Except for *M*. *arctoides*, we also detected other discordances in the positions of several macaque species among the gene trees. First, the *Alu* elements, concatenated nuclear data and mitochondrial data consistently supported a close relationship of *M*. *fuscata* and *M*. *mulatta*, while the autosomal tree (S2 Fig) suggested an outgroup clade of *M*. *fuscata* to the *sinica/arctoides* and the *fascicularis/mulatta* lineages. Tosi et al. [16] also detected a similar phylogenetic relationship of *M*. *fuscata* based on an intron sequence of the C4 gene. *M*. *fuscata* was usually considered to have diverged recently from the continental subspecies, *M*. *mulatta tcheliensis*, on the basis of fossil, morphological, and molecular evidence [4,5,76]. As an island macaque species endemic to Japan, the chance of introgression or hybridization with other macaque species is rare. Furthermore, both the X and Y chromosomal data did not show conflicting phylogenies with *Alu* elements and mitochondrial data with respect to the *M*. *fuscata*’s position. It is more likely that the incongruent position of *M*. *fuscata* relative to the autosomal tree and other gene trees resulted from differentially selected autosomal genes or ILS. Second, the Y-chromosomal tree (S2 Fig) nested *M*. *sylvanus* within the Asian macaques as a sister clade to the *sinica/arctoides* clade, which is in conflict with the *Alu* elements, concatenated nuclear data, and the mitochondrial genome based phylogenies as well as previous macaque phylogenetic studies [12,13,16,17]. *M*. *sylvanus*, the only species distributed in Africa, is isolated from the Asian macaques. Therefore, gene flow or hybridization among them can be excluded. In addition, the fossil evidence supported the earliest divergence of *M*. *sylvanus* in the genus *Macaca* [2,3]. We speculate that the conflicting position between the Y-chromosome and other genetic systems is due to insufficient informative sites of our Y-chromosomal genes or ILS. Another discordant case is that of *M*. *assamensis*. We applied PCR display methods to identify phylogenetically informative *Alu* insertions from *M*. *assamensis* for phylogenetic analysis. A total of 28 *Alu* insertions were identified and characterized. Seventeen of those loci were phylogenetically informative, which provided important information for the position of *M*. *assamensis*. *Alu* elements-based phylogeny was congruent with concatenated nuclear data and mitochondrial data, which showed support for *M*. *assamensis* and *M*. *thibetana* as the most closely related species, while the X-chromosome tree (S2 Fig) suggested *M*. *assamensis* was closer to *M*. *arctoides* than to *M*. *thibetana*. Introgression or hybridization resulted in discordant relationships with respect to *M*. *assamensis*, which cannot be rejected. However, the strong evidence from the *Alu* elements, nuclear genes, and mitochondrial genomes indicated that insufficient informative sites in the X-chromosomal data might have contributed to the discordance among gene trees.

## Supporting Information

S1 Fig The detail information on gel electrophoresis of all the *Alu* insertions is shown with *P*. *hamadryas* as outgroup. The M1 and M2 refer to 100bp ladder and 2000bp marker, respectively. The numbers 1–8 represent *P*. *hamadryas*, *M*. *sylvanus*, *M*. *leonina*, *M*. *thibetana*, *M*. *arctoides*, *M*. *fascicularis*, *M*. *fuscata*, *M*. *mulatta*, and *M*. *assamensis*, respectively.
(PDF)

S2 Fig Macaque phylogenetic relationships derived from different nuclear genes using BI, MP and ML analysis. The numbers under the branches are Bayesian posterior probabilities (BPP) and bootstrap support (BSP). Panels refer to combined autosomal sequence data (A), X chromosomal fragment (B), and Y chromosomal loci (C).
(PDF)

S3 Fig Divergence time estimations based on the Bayesian approach tree topology. Panels refer to nuclear genes (A) and mitochondrial genome (B). The numbers above branches are Bayesian posterior probabilities (BPP). The A-G besides the nodes refers to divergence times shown as in Table 2. The horizontal blue rectangles indicate the estimated 95% credibility intervals of divergence times.
(PDF)

S1 Table The recognized macaque species and species groups as classified by different authors. (DOCX)

S2 Table Information of all the species investigated in this study. (DOCX)

S3 Table All primers are employed in the present study. (DOCX)

S4 Table The information of newly idetification loci for the *Macaca* phylogenetic analysis. (XLS)

S5 Table The information of previously published identification loci for the *Macaca* phylogenetic analysis. (XLSX)

S6 Table GenBank accession number of sequence data for the nuclear dataset and the complete mitochondrial genome. (XLSX)
