# Supplementary material for: Mitochondrial Genome and Nuclear Markers Provide New Insight into the Evolutionary History of Macaques
Source: PLoS One. 2016 May 2;11(5):e0154665. doi: 10.1371/journal.pone.0154665 (PMC4852913; doi:10.1371/journal.pone.0154665)
Supplement: S1 Fig — The M1 and M2 refer to 100bp ladder and 2000bp marker, respectively. The numbers 1–8 represent P. hamadryas, M. sylvanus, M. leonina, M. thibetana, M. arctoides, M. fascicularis, M. fuscata, M. mulatta, and M. assamensis, respectively. (PDF) [file pone.0154665.s001.pdf]

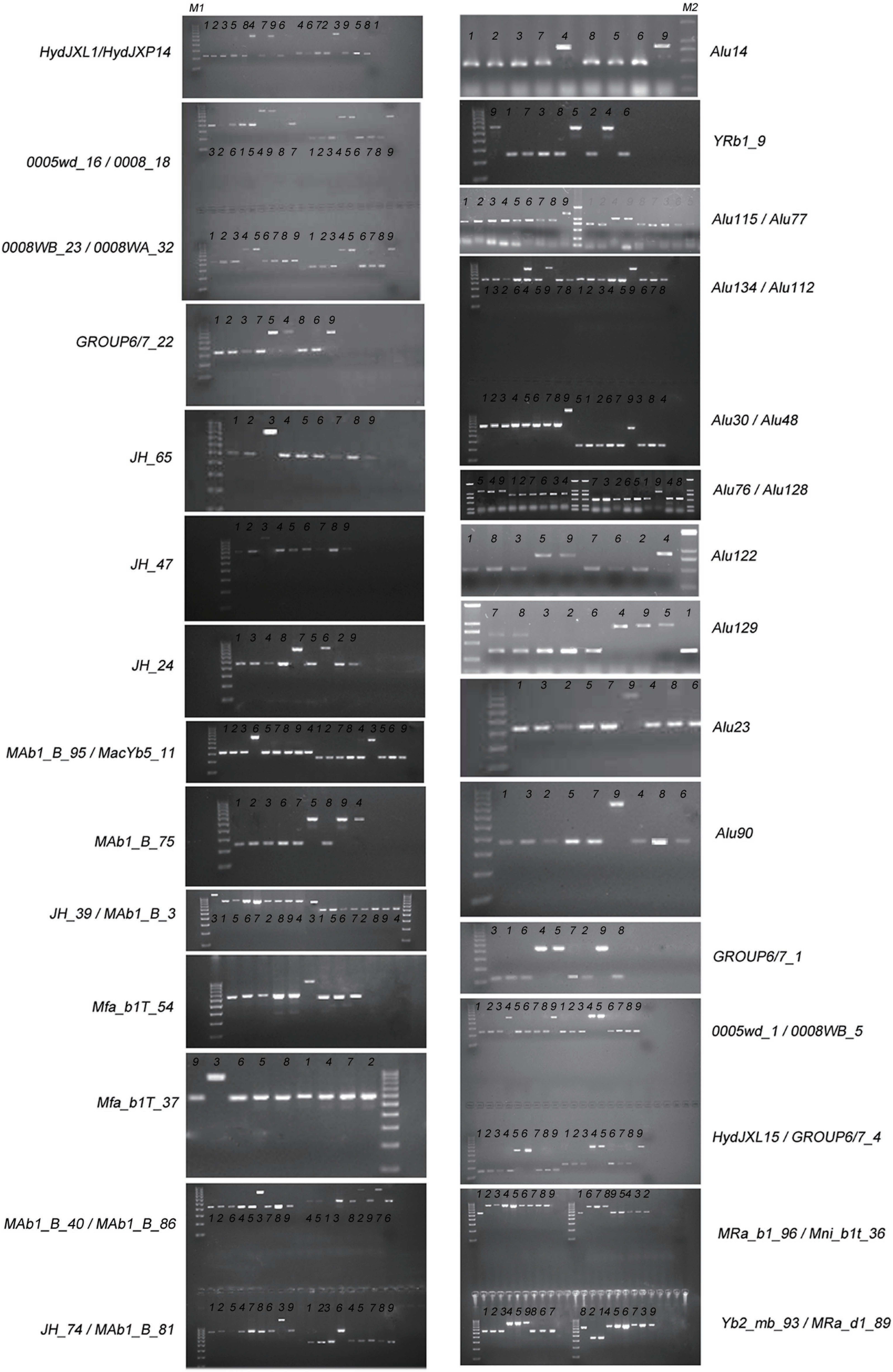
M1
M2
HydJXL1/HydJXP14
0005wd_16 / 0008_18
0008WB_23 / 0008WA_32
GROUP6/7_22
JH_65
JH_47
JH_24
MAb1_B_95 / MacYb5_11
MAb1_B_75
JH_39 / MAb1_B_3
Mfa_b1T_54
Mfa_b1T_37
MAb1_B_40 / MAb1_B_86
JH_74 / MAb1_B_81
Alu14
YRb1_9
Alu115 / Alu77
Alu134 / Alu112
Alu30 / Alu48
Alu76 / Alu128
Alu122
Alu129
Alu23
Alu90
GROUP6/7_1
0005wd_1 / 0008WB_5
HydJXL15 / GROUP6/7_4
MRa_b1_96 / Mni_b1t_36
Yb2_mb_93 / MRa_d1_89

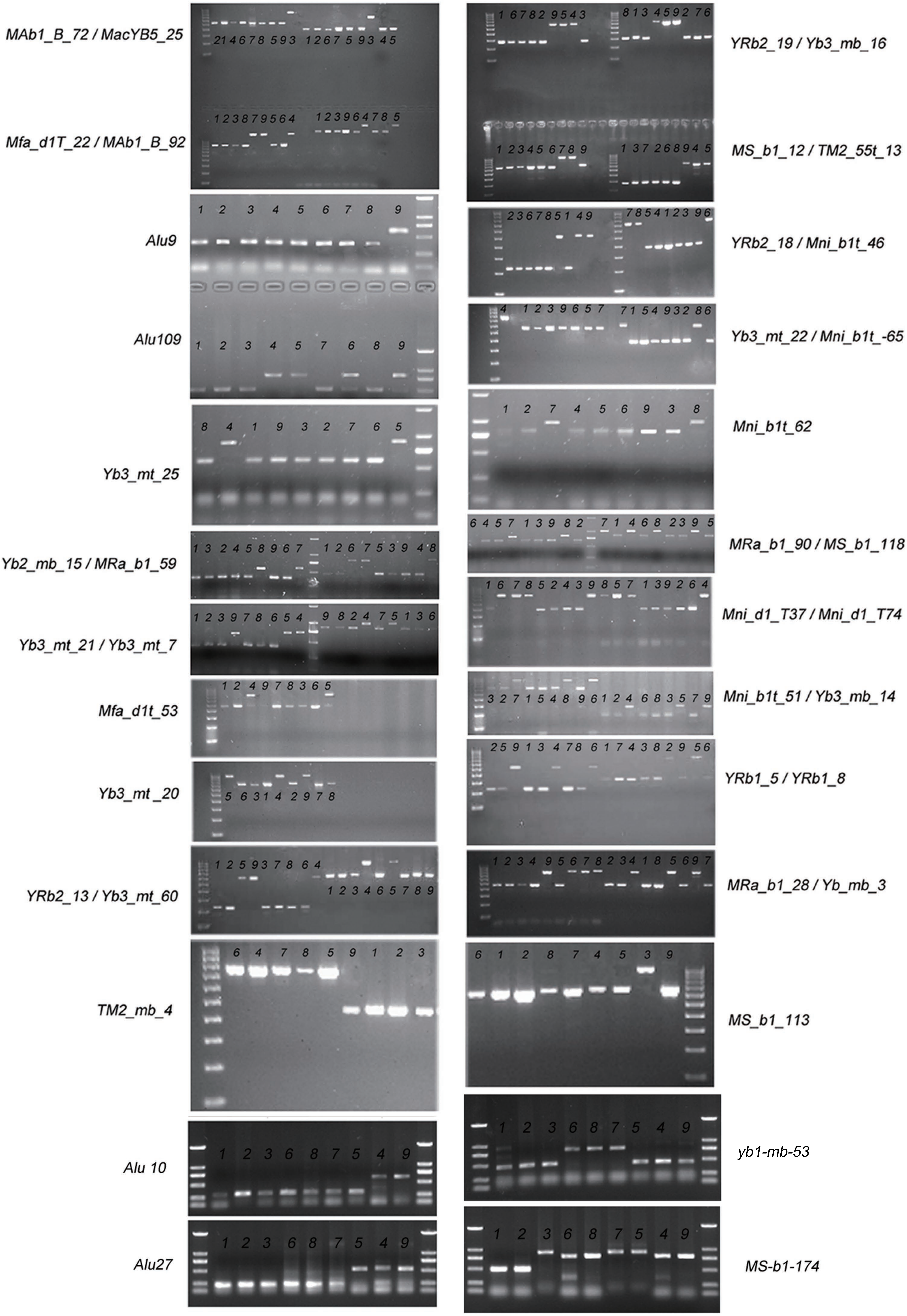
MAb1_B_72 / MacYB5_25
Mfa_d1T_22 / MAb1_B_92
Alu9
Alu109
Yb3_mt_25
Yb2_mb_15 / MRa_b1_59
Yb3_mt_21 / Yb3_mt_7
Mfa_d1t_53
Yb3_mt _20
YRb2_13 / Yb3_mt_60
TM2_mb_4
Alu 10
Alu27
YRb2_19 / Yb3_mb_16
MS_b1_12 / TM2_55t_13
YRb2_18 / Mni_b1t_46
Yb3_mt_22 / Mni_b1t_-65
Mni_b1t_62
MRa_b1_90 / MS_b1_118
Mni_d1_T37 / Mni_d1_T74
Mni_b1t_51 / Yb3_mb_14
YRb1_5 / YRb1_8
MRa_b1_28 / Yb_mb_3
MS_b1_113
yb1-mb-53
MS-b1-174
