# Supplementary figures and images for: Mitochondrial Genome and Nuclear Markers Provide New Insight into the Evolutionary History of Macaques
Source: PLoS One. 2016 May 2;11(5):e0154665. doi: 10.1371/journal.pone.0154665 (PMC4852913; doi:10.1371/journal.pone.0154665)

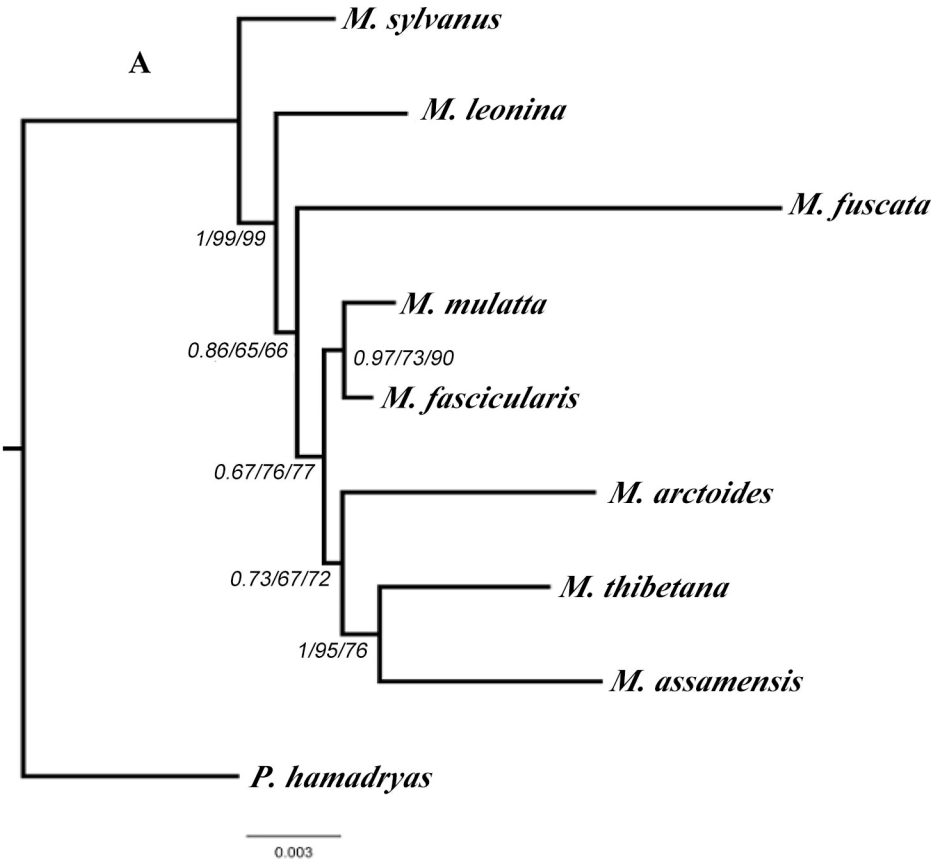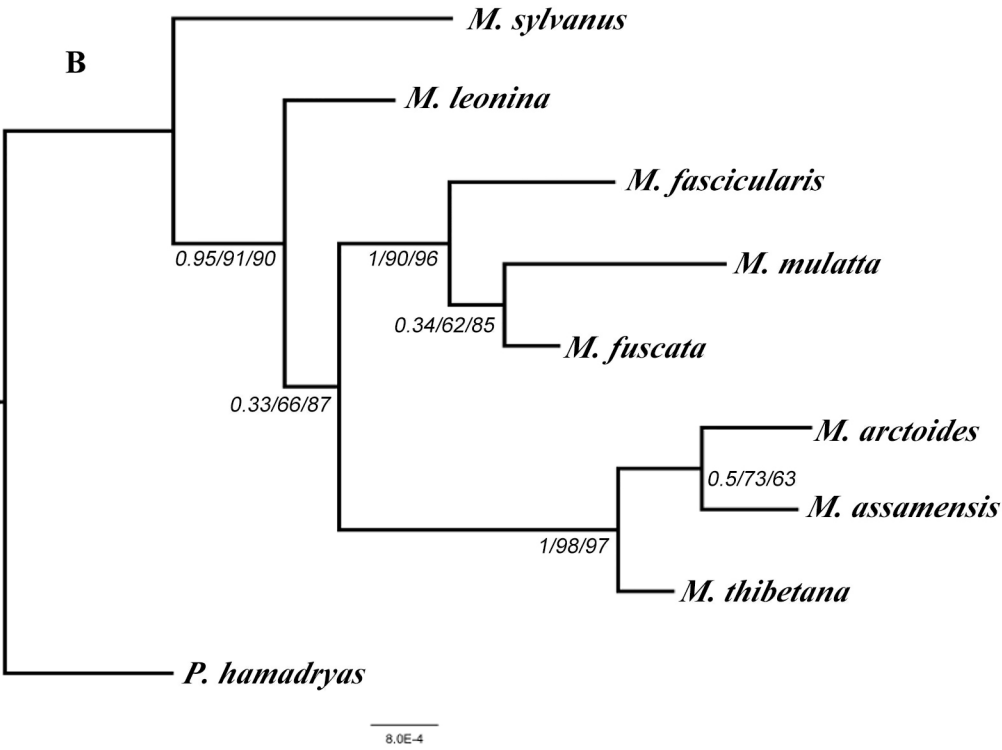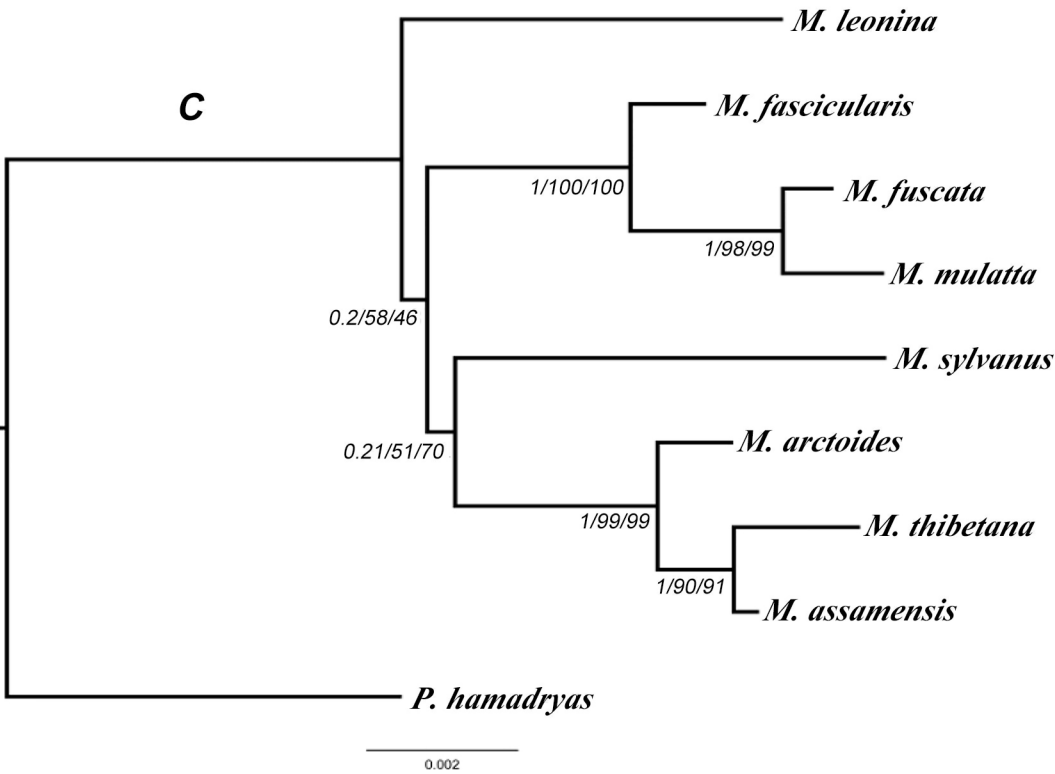

Supplement: S2 Fig — The numbers under the branches are Bayesian posterior probabilities (BPP) and bootstrap support (BSP). Panels refer to combined autosomal sequence data (A), X chromosomal fragment (B), and Y chromosomal loci (C). (PDF) [file pone.0154665.s002.pdf]

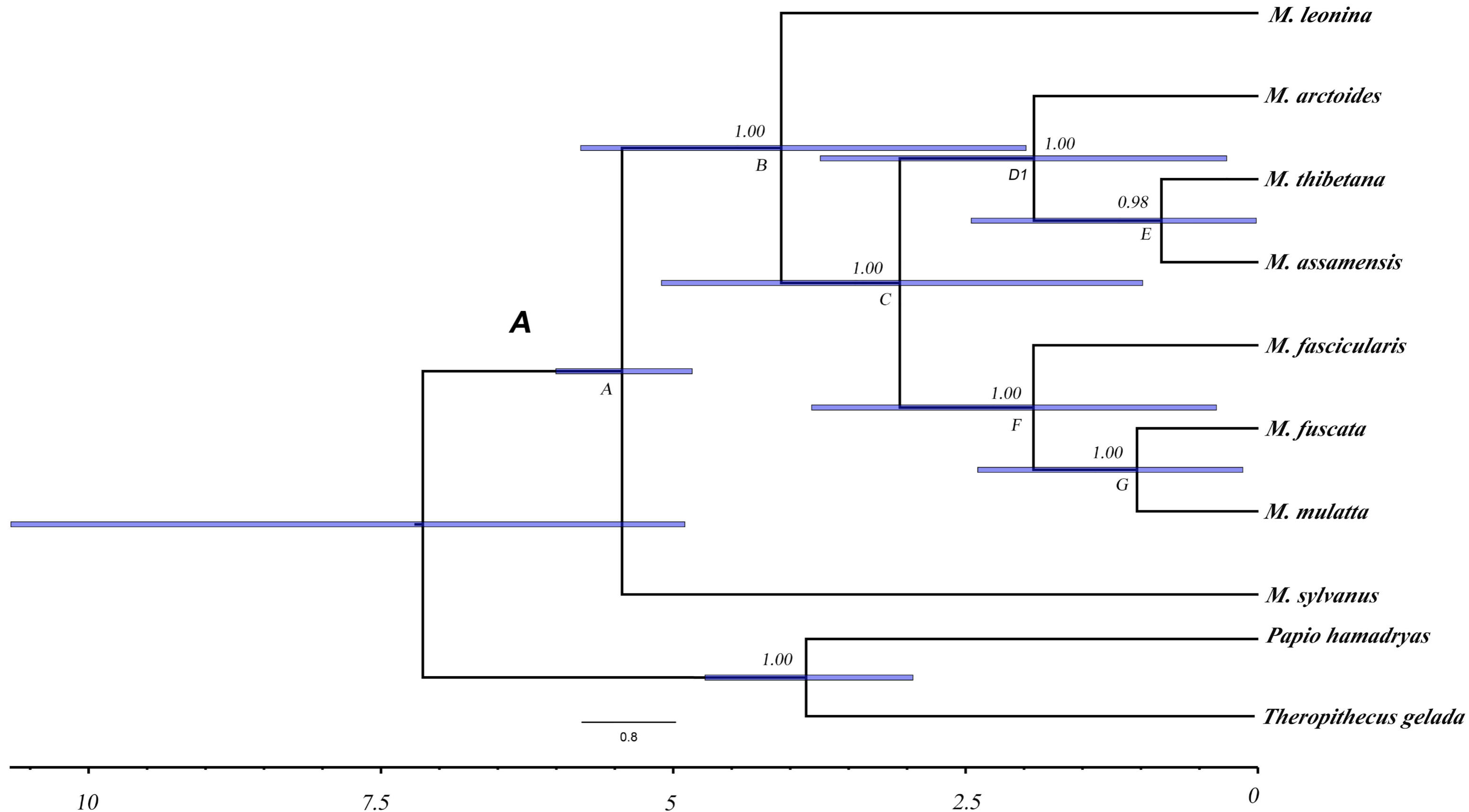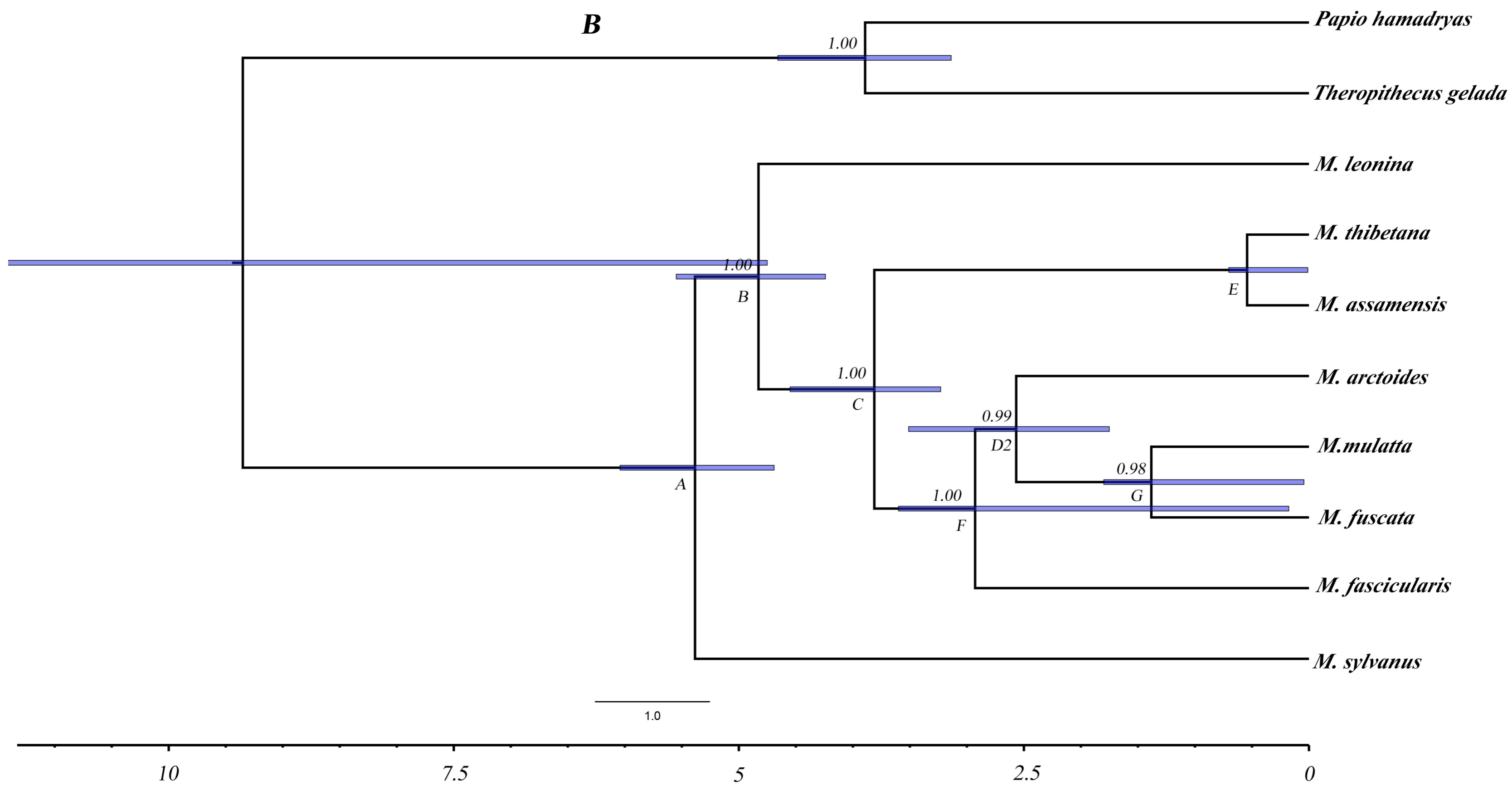

Supplement: S3 Fig — Panels refer to nuclear genes (A) and mitochondrial genome (B). The numbers above branches are Bayesian posterior probabilities (BPP). The A-G besides the nodes refers to divergence times shown as in Table 2. The horizontal blue rectangles indicate the estimated 95% credibility intervals of divergence times. (PDF) [file pone.0154665.s003.pdf]
