# Supplementary material for: Mitochondrial Genome and Nuclear Markers Provide New Insight into the Evolutionary History of Macaques
Source: PLoS One. 2016 May 2;11(5):e0154665. doi: 10.1371/journal.pone.0154665 (PMC4852913; doi:10.1371/journal.pone.0154665)
Supplement: S1 Table — (DOCX) [file pone.0154665.s004.docx]

S1 Table. The recognized macaque species and species groups as classified by different authors

| **Fooden[4]** | **Delson[2]** | **Groves[6]** | **Zinner et al. [7] and Roos et al. [8]** |
| --- | --- | --- | --- |
| ***silenus-sylvanus* group** | ***sylvanus group*** | ***sylvanus group*** | ***sylvanus group*** |
| *M. sylvanus* | *M. sylvanus* | *M. sylvanus* | *M. sylvanus* |
| *M. silenus* | ***silenus* group** | ***nemestrina group*** | ***silenus* group** |
| *M. nemestrina* | *M. silenus* | *M. silenus* | *M. silenus* |
| *M. tonkeana* | *M. nemestrina* | *M. leonina* | *M. leonina* |
| *M. maura* | *M. tonkeana* | *M. nemestrina* | *M. nemestrina* |
| *M. ochreata* | *M. maura* | *M. pagensis* | *M. pagensis* |
| *M. brunnescens* | *M. ochreata* | ***Sulawesi group*** | ***Sulawesi group*** |
| *M. hecki* | *M. brunnescens* | *M. hecki* | *M. hecki* |
| *M. nigrescens* | *M. hecki* | *M. nigra* | *M. nigra* |
| *M. nigra* | *M. nigrescens* | *M. maura* | *M. maura* |
| ***fascicularis* group** | *M. nigra* | *M. tonkeana* | *M. tonkeana* |
| *M. mulatta* | ***fascicularis* group** | *M. nigrescens* | *M. nigrescens* |
| *M. cyclopis* | *M. mulatta* | *M. ochreata* | *M. ochreata* |
| *M. fuscata* | *M. cyclopis* | *M. brunnescens* | *M. brunnescens* |
| *M. fascicularis* | *M. fuscata* | ***fascicularis* group** | ***fascicularis* group** |
| ***arctoides group*** | *M. fascicularis* | *M. fascicularis* | *M. fascicularis* |
| *M. arctoides* | ***sinica group*** | *M. arctoides* | ***arctoides* group** |
| ***sinica group*** | *M. arctoides* | ***mulatta group*** | *M. arctoides* |
| *M. radiata* | *M. radiata* | *M. mulatta* | ***mulatta group*** |
| *M. assamensis* | *M. assamensis* | *M. cyclopis* | *M. mulatta* |
| *M. sinica* | *M. sinica* | *M. fuscata* | *M. cyclopis* |
| *M. thibetana* | *M. thibetana* | ***sinica group*** | *M. fuscata* |
|  |  | *M. assamensis* | ***sinica group*** |
|  |  | *M. radiata* | *M. assamensis* |
|  |  | *M. sinica* | *M. radiata* |
|  |  | *M. thibetana* | *M. sinica* |
|  |  |  | *M. thibetana* |
|  |  |  | *M. munzula* |
