# Supplementary material for: Mitochondrial Genome and Nuclear Markers Provide New Insight into the Evolutionary History of Macaques
Source: PLoS One. 2016 May 2;11(5):e0154665. doi: 10.1371/journal.pone.0154665 (PMC4852913; doi:10.1371/journal.pone.0154665)
Supplement: S2 Table — (DOCX) [file pone.0154665.s005.docx]

S2 Table. Information of all the species investigated in this study

| **Species name** | **Common name** | **Sample origin** |
| --- | --- | --- |
| *M. sylvanus* | Barbary macaque | Chengdu Zoo, exchanged from foreign zoo |
| *M. leonina* | Northern pig-tailed macaque | Chengdu Zoo, from southwest of China |
| *M. fascicularis* | Crab-eating macaque | Hengshu Primate Center, Yibin, Sichuan province, China |
| *M. mulatta* | Rhesus macaque | Mabian county, Sichuan province, China |
| *M. fuscata* | Japanese macaque | Chengdu Zoo, exchanged from foreign zoo |
| *M. thibetana* | Tibetan macaque | Jianyang county, Sichuan province, China |
| *M. assamensis* | Assamese macaque | Chengdu zoo, from southwest of China |
| *M. arctoides* | Stump-tailed macaque | Chengdu Zoo, from Guangxi Province, China |
| *Papio hamadryas* | Hamadryas Baboon | Chengdu Zoo, exchanged from foreign zoo |
