# Supplementary material for: Mitochondrial Genome and Nuclear Markers Provide New Insight into the Evolutionary History of Macaques
Source: PLoS One. 2016 May 2;11(5):e0154665. doi: 10.1371/journal.pone.0154665 (PMC4852913; doi:10.1371/journal.pone.0154665)
Supplement: S3 Table — (DOCX) [file pone.0154665.s006.docx]

S3 Table. All primers are employed in the present study.

| **Primers for PCR-display methodology** | | | |
| --- | --- | --- | --- |
|  | | **Primer (5’-3’)** | **Reference** |
| **Linkers** | Top | TAGAAGGAGAGGACGCTGTCTGTCGAAGG | 47 |
|  | Bottom | GAGCGAATTCGTCAACATAGCATTTCTGTCCTCTCCTTC |  |
| **LNP** |  | GAATTCGTCAACATAGCATTTCT | 47 |
| **YbI** |  | GACGGAGTCTCACGCTGTT | 18 |
| **YdI** |  | GACGGAGTCTCGCTGTGTCT |  |
| **YbII** |  | AAGCTCCGCCTCCCGGGTTCC | 18 |
| **YdII** |  | GTTTCACCATGTTAGCCAGGATA |  |
| **Primers for the amplification of nuclear loci** | | | |
| **Gene** |  |  |  |
| ***ALB3*** | Albumin, intron 3 | F: GCATTCAAAGTCAACCATG | 22 |
|  |  | R: ACGAAGAGTTGCAACTGTGC |  |
| ***IRBP3*** | Interstitial retinol-binding protein, intron 3 | F: CTCTGGACACACGCCCAG | 22 |
|  |  | R: CACACTGCTGGTCAGAATGA |  |
| ***TNP2*** | Transition protein 2 | F: GCAGGTGTACAAAACCAAG | 22 |
|  |  | R: GTCTCATTAGTTGGATTTCC |  |
| ***TTR1*** | Transthyretin, intron 1 | F: GGCCCTACGGTGAGTGTT | 22 |
|  |  | R: ACTTTGACCATCAGAGGACA |  |
| ***vWF11*** | Von Willebrand Factor, intron11 | F:GAGTGCCTTGTCACTGGTCATCCCACTTCAA | 22 |
|  |  | R: GAGCTGGATGTCCTGGCCATCCATGGCAAC |  |
| ***Xq13.3*** | Xq13.3 region of the human X chromosome | F: TAGAAAAGTGTACTGGATTTG | 52 |
|  |  | R: TTAGTCAATTGGCACATAGATTC |  |
| ***SRY*** | sex-determining region, Y chromosome | F: CTTGAGAATGAATACATTGTCAGGG | 17 |
|  |  | R: CTGTGCATAAGACCATGCTGAG |  |
| ***TSPY*** | testis-specific protein, Y chromosome | F: AGCCAGGAAGGCCTTTTCTCG | 17 |
|  |  | R: CTGTGCATAAGACCATGCTGAG |  |
